# *In Vivo* Metabolism of Ibuprofen in Growing Conventional Pigs: A Pharmacokinetic Approach

**DOI:** 10.3389/fphar.2019.00712

**Published:** 2019-06-27

**Authors:** Joske Millecam, Siegrid De Baere, Siska Croubels, Mathias Devreese

**Affiliations:** Laboratory of Pharmacology and Toxicology, Department of Pharmacology, Toxicology and Biochemistry, Faculty of Veterinary Medicine, Ghent University, Merelbeke, Belgium

**Keywords:** ibuprofen, pig, metabolites, biotransformation, cytochrome P450, uridine 5′-diphospho-glucuronosyltransferase, *in vivo*, age

## Abstract

The juvenile conventional pig has been suggested as a preclinical animal model to evaluate pharmacokinetic (PK), pharmacodynamic (PD), and safety parameters in children. However, a lot of developmental changes in pig physiology still need to be unraveled. While the *in vitro* ontogeny of pig biotransformation enzymes is getting more attention in literature, the *in vivo* developmental changes have not yet been investigated. Therefore, the aim of the current study was to evaluate the biotransformation of ibuprofen (IBU) in conventional pigs aged 1 week, 4 weeks, 8 weeks, and 6–7 months after a single intravenous and oral administration of 5 mg/kg body weight (BW) of IBU, using a PK approach in a crossover design for each age group. An ultra-performance liquid chromatography–tandem mass spectrometry (UPLC-MS/MS) method was developed and validated to determine 2-hydroxyibuprofen (2OH-IBU), carboxyibuprofen (COOH-IBU), and ibuprofen glucuronide (IBU-GlcA) in pig plasma. All three metabolites could be quantified in plasma and the following PK parameters were determined: *C*_max_, *T*_max_, AUC_0→6h_, area under plasma concentration–time curve (AUC) ratio between parent drug and metabolite, and the absolute oral bioavailability of the parent drug IBU. The plasma concentrations of the metabolites were always lower than those of IBU. The bioavailability was high, indicating limited pre-systemic biotransformation. The AUC ratio of 2OH-IBU and COOH-IBU/IBU showed a significant increase at 4 weeks of age compared to the 1-week-old and 6- to 7-month-old pigs. Interestingly, the IBU-GlcA/IBU AUC ratio did not change with age. The present study demonstrated that the main metabolites of IBU in human are also present in growing pigs. The oxidative phase I metabolism of IBU in growing conventional pigs did change with age. In contrast, age did not seem to affect the glucuronidation capacity of IBU in conventional pigs, although more studies with other substrate drugs are needed to confirm this.

## Introduction

Nowadays, the pig is being generally accepted as a preclinical animal model to predict the pharmacokinetics (PK), pharmacodynamics (PD), and safety of a drug. This is attributed to the great anatomical and physiological similarities with human regarding the organs involved in absorption, distribution, metabolism, and excretion (ADME) of a drug (Swindle et al., 2012; Helke and Swindle, 2013). For example, Henze et al. (2018) demonstrated a favorable role for the pig over the dog to predict human oral bioavailability. Yoshimatsu et al. (2016) found the NIBS (Nippon Institute for Biological Science) minipig to be superior to mouse and rat for the prediction of human PK of 14 different drugs. Recently, the juvenile pig has been suggested as a possible animal model for the child. Several studies have already demonstrated the good similarities in PK characteristics between the piglet and the child (Roth et al., 2013; Gasthuys et al., 2018). Nevertheless, comparative data between human and pig are still largely lacking for the ADME processes, especially in the pediatric population.

Recently, some efforts were made to unravel the ontogeny of the physiological processes involved in the ADME characteristics of selected drugs in pigs. Regarding renal excretion, Gasthuys et al. (2017) demonstrated a comparable glomerular filtration rate (GFR) between the growing conventional pig and human. The metabolism of adult pigs has already been extensively studied, but data regarding developmental changes in pig metabolism are much more limited (Skaanild, 2006;Puccinelli et al., 2011). Hu (2015) and Millecam et al., (2018) both observed an increase in *in vitro* biotransformation rate with age for substrates metabolized by phase I cytochrome P450 (CYP) enzymes, in accordance with human *in vitro* developmental data. This was due to an increase in the absolute amount of CYP proteins in the pig microsomes as was demonstrated in growing piglets (Millecam et al., 2018). Regarding phase II enzymes, to the authors’ knowledge, Van Peer et al. (2017) were the only ones to evaluate developmental differences in phase II metabolism in the Göttingen minipig. Using the UGT-Glo™ assay, an increase in microsomal uridine diphosphate glucuronosyltransferase (UGT) activity with age, from 1 week old onwards, was observed. In fetal microsomes and microsomes from pigs aged 1 and 3 days, no UGT activity could be observed. However, using a human UGT1A1 antibody, pig UGT analogs were immunohistochemically detected in liver slices, even in the fetal samples. The adult minipig UGT activity was higher compared to that observed in adult human liver microsomes (Van Peer et al., 2017). Next to an effect of age, other factors may also influence drug-metabolizing enzymes such as breed, gender, diet, epigenetic factors, transcriptional regulation, and circadian variation (Helke et al., 2016). Consequently, these factors need to be taken into account when one wants to make human predictions based on data obtained in pigs. The current literature on the ontogeny of biotransformation in pigs is mainly based on *in vitro* experiments. *In vivo* evaluation of the maturation of drug-metabolizing processes in pigs has not yet been performed. Yet, these types of studies could provide crucial information that would improve scaling of results from pig to child.

Ibuprofen (IBU) is one of the most widely used analgesic, anti-pyretic, and anti-inflammatory drugs, both in adults and in children, due to its good tolerability compared to other non-steroidal anti-inflammatory drugs (NSAIDs). IBU is a colorless, crystalline stable solid with a water solubility of 21 mg/L (25°C) and a log octanol-water partition coefficient of 3.97 (DrugBank). The relatively fast absorption from the upper gastrointestinal tract, when administered as suspension, is followed by extensive metabolism and clearance *via* the kidneys, leading to a short plasma elimination half-life (Rainsford, 2009). Oxidative phase I metabolism in humans is facilitated by CYP2C8, CYP2C9, and CYP2C19. Phase II biotransformation is mainly performed by UGTs leading to acyl glucuronides of IBU (IBU-GlcA) and its phase I metabolites (Rainsford, 2009). Both Tan et al. (2003) and Rudy et al. (1991) recovered about 70% of the racemic dose in 24-h urine of adults as IBU, 2-hydroxyibuprofen (2OH-IBU), and carboxyibuprofen (COOH-IBU), whether or not glucuronidated. Measurement of IBU metabolites in plasma was not performed in these studies. No literature evaluating *in vivo* biotransformation of IBU in children is available. Nevertheless, the *in vitro* available data suggest an increase of both involved CYPs and UGTs with age, suggesting a simultaneous increasing clearance (Lu and Rosenbaum, 2014). Human CYPs show high homology with their pig counterparts, suggesting that the homologous CYPs could be involved in the biotransformation of the same drug (Puccinelli et al., 2011; Millecam et al., 2018). The same is true for the phase II UGT enzymes with at least 70% amino acid homology between human and pig UGT variant (Vaessen et al., 2017). The growing conventional pig could thus possibly be a good animal model to evaluate *in vivo* changes in drug-metabolizing properties due to the presence of homologous CYP enzymes and UGT enzymes.

The current study aims to evaluate developmental changes in the biotransformation of IBU in the conventional pig. Therefore, single bolus plasma PK studies, using intravenous (IV) and oral (PO) dosing in a crossover design, were performed in growing conventional pigs of four age categories. IBU, 2OH-IBU, COOH-IBU, and IBU-GlcA were included in the study.

## Materials and Methods

### Animals and Experimental Design

Care and use of the animals were in full compliance with the national and European legislation on animal welfare and ethics, and the study was approved by the ethical committee of the Faculties of Veterinary Medicine and Bioscience Engineering of Ghent University (EC2016/105) (Anonymous, 2010;Flemish Government, 2017). Four age groups were included in the study, corresponding to the main age groups of the human pediatric population, namely, neonate, infant, child, and adolescent (Gad, 2015;Gasthuys et al., 2016). One-week-old piglets (3.0 ± 0.4 kg BW; Landrace × Large White × Maximus, RA-SE Genetics, Merkem, Belgium), 4-week-old piglets (7.0 ± 0.8 kg BW; Maximus, RA-SE Genetics, Bassilly, Belgium), 8-week-old piglets (20.1 ± 3.4 kg BW; Landrace × Large White, RA-SE Genetics and Convis, Ettelbruck, Luxembourg), and 6- to 7-month-old pigs (134.0 ± 4.6 kg BW for males and 142.0 ± 9.8 kg for females; Landrace × Large White, RA-SE Genetics and Convis, Ettelbruck, Luxembourg) were used, representing the latter human age groups, respectively. Each age group in the PK study consisted of 8 pigs (4 ♂/4 ♀). All male pigs were intact. Since male and female pigs reach puberty at different ages and the influence of sex hormones on the PK of IBU was of interest, all 4 male pigs were 6 months old, while the 4 female pigs were 7 months old (Van den Broeke et al., 2015).

A double-lumen catheter in the jugular vein and a gastrostomy button was inserted to facilitate blood sampling and IV and PO dosing, as described elsewhere (Gasthuys et al., 2009; Gasthuys et al., 2017). Respecting 1-day recovery, all pigs received an IV bolus of 5 mg/kg BW IBU (Ibuprofenum, Fagron Inc., Meer, Belgium), which was dissolved in 0.9% NaCl (stock solution of 100 mg/ml) and administered through the proximal lumen of the jugular catheter. Blood (1 ml) was taken from the distal lumen of the jugular catheter prior to and at 5, 10, 20, 30, 45, 60 min and 1.5, 2, 2.5, 3, 4, and 6 h after administration (p.a.). After 1 day washout, all pigs received an oral dose of a pediatric IBU suspension (5 mg/kg BW; Ibuprofen EG^®^ 40 mg/ml, Eurogenerics, Brussels, Belgium) through the stomach button. Blood samples (1 ml) were again drawn from the distal lumen of the catheter prior to and at 5, 10, 20, 30, 45, and 60 min and 1.25, 1.5, 1.75, 2, 2.5, 3, 4, and 6 h p.a. All blood samples were drawn into 4-ml K_3_EDTA collection tubes (Vacutest^®^, Piove di Sacco, Kima, Italy), immediately kept on ice, and centrifuged for 10 min at 2,095 *g* (Beckman Coulter Allegra X-15R, Brea, California, USA). The plasma was aliquoted, frozen, and stored at <−15°C until analysis. At the end of the study, all pigs were euthanized by IV injection of an overdose of pentobarbital (Sodium pentobarbital 20%^®^, Kela, Hoogstraten, Belgium). When the double-lumen catheter was no longer functional, euthanasia was performed by intramuscular injection with a mixture (1:1, 0.22 ml/kg) of xylazine hydrochloride (Xyl-M 2%^®^, VMD, Arendonk, Belgium) and tiletamine-zolazepam (Zoletil 100^®^, Virbac, The Netherlands), followed by intracardiac injection of an overdose of pentobarbital.

### Determination of IBU, 2OH-IBU, COOH-IBU, and IBU-GlcA in Pig Plasma

**Figure 1:**
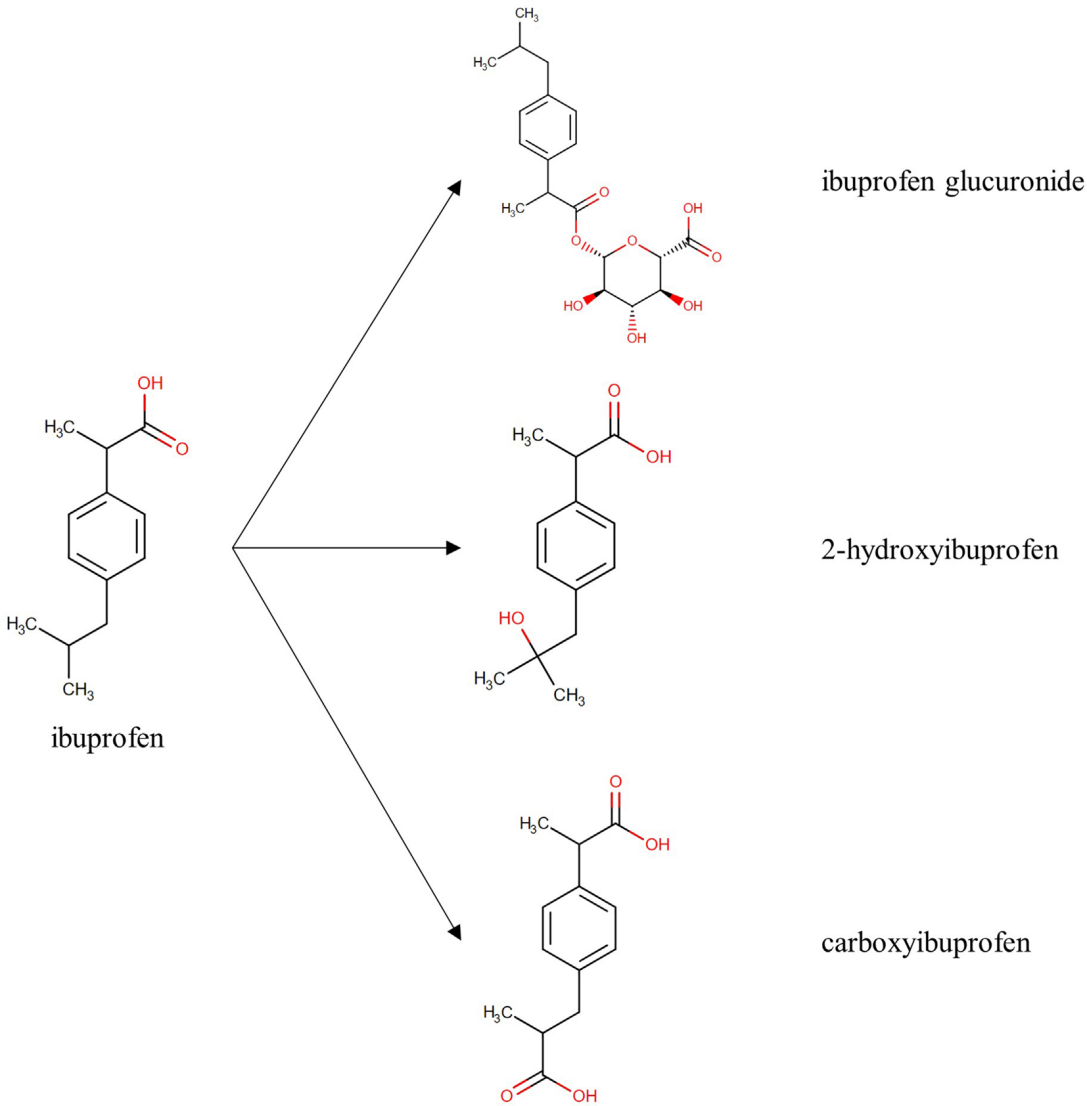
Chemical structure of ibuprofen and its metabolites, ibuprofen glucuronide, 2-hydroxyibuprofen, and carboxyibuprofen. Structures are derived from Drugbank^1^. (Wishart et al., 2008)

The total IBU concentrations were determined as the sum of R- and S-IBU using a validated ultra performance liquid chromatography with an ultraviolet detector (UPLC-UV) method according to Millecam et al. (2019). IBU metabolites were quantified using an in-house developed and validated ultra-performance liquid chromatography–tandem mass spectrometry (UPLC-MS/MS) method. Stock solutions of 1 mg/ml of 2OH-IBU (Toronto Research Chemicals, Ontario, Canada), COOH-IBU (Sigma-Aldrich, Overijse, Belgium), ibuprofen acyl-β-D-glucuronide (IBU-GlcA; Sigma-Aldrich), and the internal standard (IS) 2-hydroxyibuprofen-d6 (2OH-IBUd6; Toronto Research Chemicals) were prepared in methanol (Biosolve BV, Valkenswaard, The Netherlands) and stored at ≤−75°C. The chemical structures of IBU, 2OH-IBU, COOH-IBU, and IBU-GlcA are shown in **Figure 1**. Working solutions with a mix of 2OH-IBU, COOH-IBU, and IBU-GlcA were prepared weekly in methanol and stored at ≤−75°C. A working solution of 0.5 µg/ml in methanol for the IS was prepared and stored at ≤−75°C. All solvents used for the preparation of the stock and working solutions and for the analytical instrument were of ULC/MS grade.

The sample preparation consisted of a liquid–liquid extraction. After thawing the plasma, 250 µl was transferred into a 15-ml falcon tube. Next, 25 µl of the IS working solution, 250 µl of methanol, 250 µl of Milli-Q water, 250 µl of HCl 1N (p.a. grade, Thermo Fisher Scientific, Merelbeke, Belgium) in water, and 5 ml of ethyl acetate (p.a. grade, Merck, Darmstadt, Germany) were added with intermittent vortex-mixing. After 20 min of gentle rolling, the samples were centrifuged for 5 min at 3,724 *g* and 4°C. The organic phase (4.5 ml) was transferred to a glass tube and evaporated under nitrogen at 40°C. The dry residue was dissolved in 200 µl of 75/25 Milli-Q water/acetonitrile (ACN, Biosolve), filtered through a Millex-GV polyvinylidene flouride (PVDF) filter (0.22 µm, Merck), and brought in a conical autosampler vial (Filterservice, Eupen, Belgium). A 5-µl aliquot was injected onto the UPLC-MS/MS instrument.

Chromatographic separation was achieved on an Acquity UPLC^®^ BEH C18 column (50 × 2.1 mm i.d., dp: 1.7 µm; Waters, Zellik, Belgium) combined with a Vanguard pre-column of the same type (5 × 2.1 mm i.d., dp: 1.7 µm; Waters). Mobile phase A consisted of 0.1% acetic acid (Merck) in Milli-Q water and mobile phase B consisted of 0.1% acetic acid in ACN. The following gradient was run: 0–2.5 min (75% A, 25% B), 2.5–3.5 min (linear gradient to 90% B), 3.5–5.4 min (10% A, 90% B), 5.4–5.5 min (linear gradient to 75% A), and 5.5–7 min (75% A, 25% B). The flow rate was set at 0.4 ml/min. The column and autosampler temperature were set at 30°C and 10°C, respectively.

The UPLC column effluent was interfaced to a Quattro Premier XE triple quadrupole mass spectrometer (MS/MS), equipped with an electrospray ionization (ESI) probe operating in the negative ionization mode (both from Waters). The following parameters were used: capillary voltage: 3.2 kV; cone voltage: 25 V for 2OH-IBU and IBU-GlcA, and 20 V for 2OH-IBUd6 and COOH-IBU; source temperature: 120°C; desolvation temperature: 350°C; cone gas flow: 20 L/h; desolvation gas flow: 750 L/h; and collision gas flow: 0.03 ml/min. MS/MS acquisition was performed in the multiple reaction monitoring (MRM) mode. The following precursor ion → product ion transitions were used for identification and quantification, respectively: COOH-IBU: mass-to-charge ratio (*m/z*) 235.1 → 191.2 [collision energy (CE) 10 eV] and *m/z* 235.1 → 72.9 (CE 15 eV), IBU-GlcA: *m/z* 381.2 → 193.0 (CE 10 eV) and *m/z* 381.2 → 112.9 (CE 15 eV). For 2OH-IBU and 2OH-IBUd6, only one precursor ion → product ion transition could be observed in the MS/MS spectrum, i.e., 2OH-IBU: *m/z* 221.4 → 177.2 (CE 8 eV), 2OH-IBUd6: *m/z* 227.1 → 183.3 (CE 9 eV).

The described UPLC-MS/MS method for the determination of IBU metabolites in pig plasma was validated according to European and international guidelines and recommendations (Knecht and Stork, 1974; European Commission, 2002; US Department of Health and Human Services, 2015). The following parameters were evaluated: linearity [correlation coefficient (*r*) and goodness-of-fit (gof)], within-run and between-run precision and accuracy, limit of quantification (LOQ), limit of detection (LOD), carry-over, and stability of the analytes of interest in sample extracts during storage in the autosampler (temperature: 10°C).

### Pharmacokinetic Analysis

The areas under the plasma concentration–time profiles from time zero to 6 h (AUC_0→6h_) were determined for 2OH-IBU, COOH-IBU, IBU-GlcA, and IBU. The linear up log down trapezoidal rule was applied, and concentrations below the corresponding LOQ were excluded. Next, the ratios of the AUC_0→6h_ of each metabolite to the AUC_0→6h_ of IBU were calculated. The observed maximum plasma concentrations (*C*_max_) and the observed time at which *C*_max_ was reached (*T*_max_) were reported. The absolute oral bioavailability (*F*) for IBU was calculated as the ratio between AUC_0→6h, PO_ and AUC_0→6h, IV_. The non-compartment analysis (NCA) package of the software Phoenix version 8.1 (Certara, Princeton, New Jersey, USA) was used.

### Statistical Analysis

A one-way nested ANOVA was performed to evaluate possible significant (*p* < 0.05) effects of age and gender on *C*_max_, *T*_max_, AUC_0→6h_, AUC ratio, and *F*. A pairwise *t* test was performed to determine significant (*p* < 0.05) differences between IV and PO administration regarding *T*_max_, *C*_max_, AUC_0→6h_, and the AUC ratio of the three metabolites and IBU. Finally, a two-way ANOVA was performed to evaluate age-related changes between the different metabolites. Post hoc analysis was done using Tukey’s HSD (Honestly Significant Difference) test and homogeneity of variance was tested using Levene’s test (*p* > 0.01). If the assumption of homogeneity was not met, log transformation of data was performed. All statistical analyses were performed in RStudio version 1.1.456 (RStudio Inc., Boston, MA, USA).

## Results

### UPLC-MS/MS Method for IBU Metabolites

The internal standard 2OH-IBUd6 was used for the quantification of 2OH-IBU and COOH-IBU. The quantification of IBU-GlcA was done without internal standard as the IS eluted several minutes before IBU-GlcA, resulting in non-linear peak-area ratios. Initially, fenoprofen was tested as IS for the IBU-GlcA analysis, but results were not acceptable (data not shown). The validation results are shown in **Supplementary Table S1** and **S2**. For all three components, linear calibration curves were obtained covering a range of 20–3,000 ng/ml for 2OH-IBU and COOH-IBU and a range of 10–600 ng/ml for IBU-GlcA. Good correlation between analyte concentrations and detected responses was demonstrated for all three compounds, which was reflected in good correlation coefficients (*r*) that were higher than 0.99 and gof coefficients ranging between 3.16% and 5.67%, which were lower than the maximum acceptance criterion of 10%. The acceptance criteria outlined in the European and international guidelines and recommendations for within- and between-run accuracy and precision were met for all compounds at three defined concentration levels (200, 750, and 2,000 ng/ml for 2OH-IBU and COOH-IBU and 40, 150, and 400 ng/ml for IBU-GlcA) (Knecht and Stork, 1974; European Commission, 2002; US Department of Health and Human Services, 2015). The LOQs were 20 ng/ml for 2OH-IBU and COOH-IBU and 10 ng/ml for IBU-GlcA. Both 2OH-IBU and COOH-IBU had the same LOD of 0.59 ng/ml, whereas IBU-GlcA had an LOD of 0.27 ng/ml. No interfering peaks could be detected in any of the blank samples at the retention times of the analytes of interest, which means that the method was specific. Finally, no carry-over was detected as there was no analyte detected in the solvent sample injected after the highest calibrator or quality control. An illustration of the obtained chromatograms is demonstrated in **Supplementary Figure S1**. The IBU metabolites were stable in extracted samples, stored in the autosampler at a temperature of 10°C for 24 h. Overall, the developed UPLC-MS/MS method was proven to be robust and selective for the high-throughput analysis of IBU metabolites in plasma.

### Pharmacokinetic Analysis

**Figure 2:**
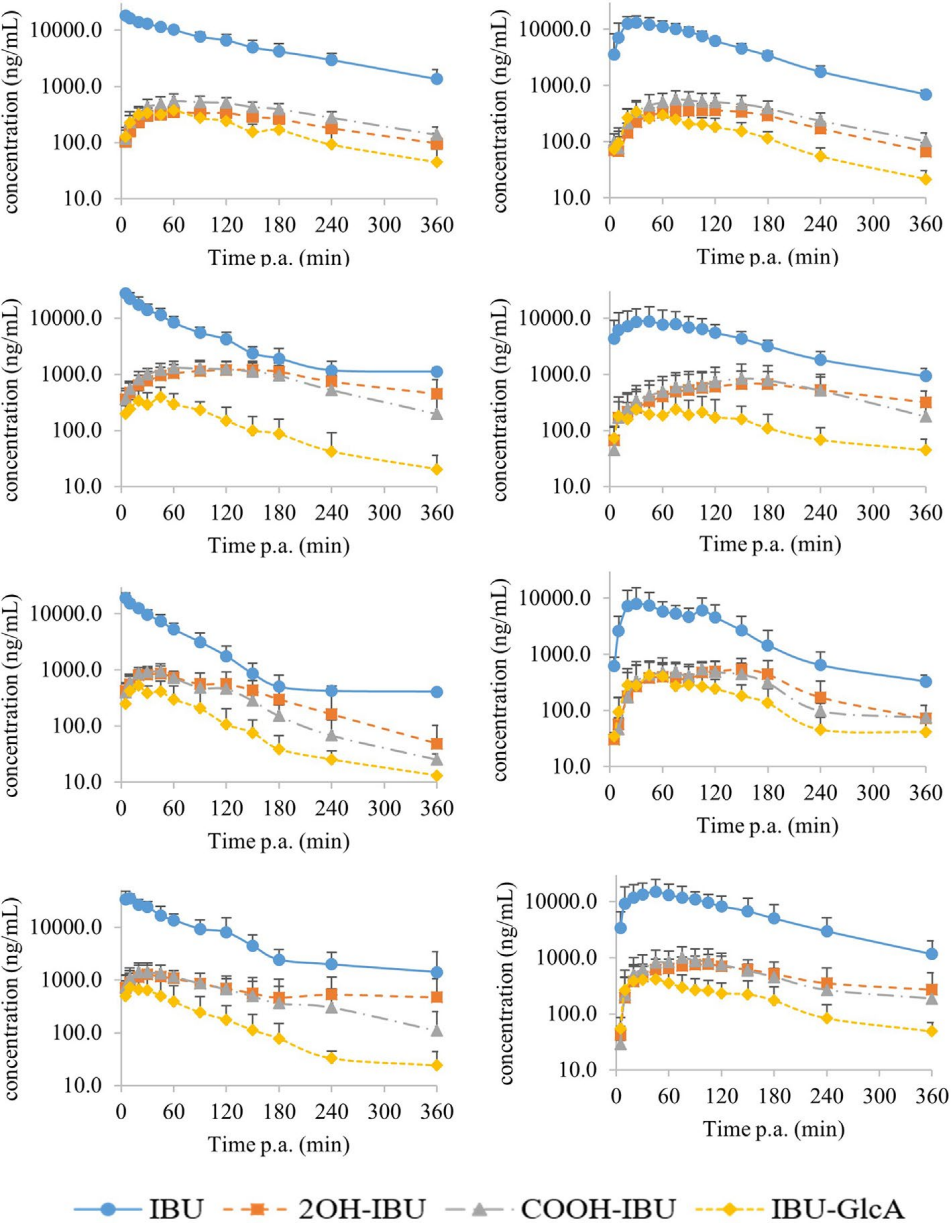
The mean (±SD) log concentration–time profiles after intravenous (IV) (left) and oral (PO) (right) administration of 5 mg/kg BW of ibuprofen (blue dots) with their corresponding metabolites 2-hydroxyibuprofen (2OH-IBU, orange square), carboxyibuprofen (COOH-IBU, gray triangle), and ibuprofen glucuronide (IBU-GlcA, yellow diamond). From top to bottom are the curves for the 1-week-old, 4-week-old, 8-week-old, and 6- to 7-month-old pigs (each time, 8 pigs, 4 ♂, 4 ♀) represented, respectively. Time post administration (p.a.).

The mean (±standard deviation, SD) plasma concentration–time profiles of the three determined metabolites and IBU are depicted in **Figure 2** for all four age groups and both ways of administration. The plasma concentrations of 2OH-IBU, COOH-IBU, and IBU-GlcA were always lower than that of IBU. The *C*_max_, *T*_max_, AUC, and AUC ratio results can be found in **Table 1** as well as *F* for IBU.

**Table 1:** The mean values of the pharmacokinetic parameters (standard deviation, SD) of ibuprofen and its main metabolites, 2-hydroxyibuprofen, carboxyibuprofen, and ibuprofen glucuronide after intravenous (IV) or oral (PO) administration of 5 mg/kg BW ibuprofen to pigs aged 1 week, 4 weeks, 8 weeks, and 6–7 months (each time, 8 pigs, 4 ♂, 4 ♀).

	1 week old		4 weeks old		8 weeks old		6–7 months old	
	IV	PO	IV	PO	IV	PO	IV	PO
*Ibuprofen*								
*C*_0_/*C*_max_ (µg/ml)	18.0 (1.3)^a$^	13.6 (4.0)^$^	26.5 (10.7)^a$^	11.4 (7.4)^$^	18.2 (4.4)^a$^	11.9 (4.9)^$^	39.4 (6.2)^b$^	16.8 (9.0)^$^
*T*_max_ (min)	–	32.3 (20.5)	–	72.5 (57.7)	–	62.5 (40.4)	–	63.8 (50.6)
AUC_0–6h_ (min*µg/ml)	1,847.5 (449.0)^a^	1,673.2 (358.6)^ab^	1,702.1 (495.4)^a^	1,393.9 (472.7)^ab^	990.6 (195.7)^b^	891.2 (386.8)^a^	2,603.5 (937.1)^c^*	1,971.5 (943.2)^b^
F (%)		94.8 (25.2)		85.5 (30.9)		90.1 (33.9)		84.4 (41.7)
*2-hydroxyibuprofen*								
*C*_max_ (ng/ml)	384.2 (39.2)^a^	397.4 (128.7)^a^	1,282.3 (528.8)^bA$^	746.2 (419.9)^abAB$^	934.7 (266.0)^ab^	713.8 (334.3)^ab^	1,483.3 (737.1)^bA^	870.5 (374.1)^b^
*T*_max_ (min)	80.6 (33.0)^ab^	97.5 (27.8)^a^	124.3 (40.36)^aA^	195.0 (107.9)^b^	46.3 (32.81)^b$^	110.6 (60.0)^ab$^	63.8 (56.1)^b^	101.9 (53.1)^a^
AUC_0–6h_ (min*µg/ml)	82.2 (9.1)^a^	78.8 (20.8)^AB^	320.3 (135.0)^bA$^	167.8 (115.2)^A$^	127.7 (65.9)^aA^	96.8 (45.3)	209.5 (143.5)^abA^	160.8 (78.4)^A^
Ratio AUC (%)	4.7 (1.2)^a^	4.8 (1.09)^a^	17.6 (12.6)^bA^	11.6 (5.6)^bA^	12.8 (6.0)^abA^	11.3 (4.0)^b^	8.7 (5.8)^a^	9.8 (6.4)^abAB^
*Carboxyibuprofen*								
*C*_max_ (ng/ml)	594.7 (171.9)^a^	604.0 (241.8)	1,384.5 (464.9)^bA^	913.4 (648.4)^A^	1,005.0 (289.5)^ab^	694.9 (328.0)	1,601.6 (593.6)^cA^	1,118.2 (552.9)
*T*_max_ (min)	84.4 (32.0)^a^	91.9 (24.6)	90.0 (24.5)^aAB$^	150.0 (60.0)^$^	34.4 (9.4)^b$^	101.3 (43.7)^$^	38.8 (22.8)^b$^	101.9 (55.5)^$^
AUC_0–6h_ (min*µg/ml)	124.5 (20.1)^a^	113.8 (40.6)^A^	284.7 (124.7)^bA^	175.8 (126.8)^A^	106.0 (33.1)^aA^	98.0 (36.6)	190.2 (96.5)^abA^	160.3 (61.9)^A^
Ratio AUC (%)	7.3 (2.6)^a^	6.7 (1.8)	15.3 (10.7)^bA^	11.5 (4.2)^A^	10.9 (3.4)^abAB^	7.7 (5.6)	7.8 (4.0)^a^	10.5 (8.3)^A^
*Ibuprofen glucuronide*								
*C*_max_ (ng/ml)	404.3 (92.9)	370.0 (118.1)	393.1 (156.5)^B^	335.4 (238.8)^B^	658.1 (357.0)	589.7 (335.3)	818.9 (695.8)^B*^	508.6 (302.2)
*T*_max_ (min)	51.9 (23.9)^a^	45.0 (21.2)	37.1 (10.4)^abB$^	93.1 (51.7)^$^	20.6 (12.1)^b$^	69.4 (39.2)^$^	29.4 (27.8)^ab^	69.3 (60.0)
AUC_0–6h_ (min*µg/ml)	61.0 (15.2)^$^	44.4 (12.6)^B$^	41.2 (20.4)^B^	40.9 (27.0)^B^	42.9 (22.8)^B^	58.1 (21.6)	63.3 (55.7)^B^	60.5 (34.7)^B^
Ratio AUC (%)	3.5 (1.1)	2.7 (2.8)^a^	2.1 (1.1)^B^	2.8 (1.3)^aB^	4.2 (1.8)^B$^	5.5 (2.8)^b$^	2.9 (2.7)^*^	3.7 (1.9)^aB^

C_0_, plasma concentration at time zero after intravenous (IV) administration for ibuprofen; C_max_, maximum plasma concentration; T_max_, time at which the C_max_ is observed; AUC, area under plasma concentration–time curve; ratio AUC, ratio of AUC of metabolite to the AUC of ibuprofen.

Significant differences for a PK parameter between the age groups and within administration route are indicated with different small letters. Significant sex differences are indicated with an asterisk (*). Significant differences between IV and PO for the PK parameters, except C_0_/C_max_ of IBU, are indicated with a $. Significant differences between the different metabolites within every age group and administration route are indicated with different capital letters.

Significant sex differences were only observed after IV administration in the 6- to 7-month-old pigs. The AUC of IBU was significantly higher in the males compared to the females. Consequently, the AUC ratio of IBU-GlcA was significantly lower for the males since no sex differences were observed in the AUC of the GlcA. Next to that, *C*_max_ of IBU-GlcA was significantly higher in the 6- to 7-month-old females compared to the males.

AUC and *C*_max_ of the metabolites were always lower, while *T*_max_ of the metabolites was always later compared to those of IBU after both IV and PO administration. Significant age differences, as summarized in **Table 1**, were observed for C_0,IBU_, AUC_IBU_, *T*_max,IBU-GlcA_, and all PK parameters of 2OH-IBU and COOH-IBU after IV administration. After PO administration, significant age differences were observed for AUC_IBU_, *C*_max,2OH-IBU_, *T*_max,2OH-IBU_, and AUC ratio of 2OH-IBU and IBU-GlcA. No significant age differences were observed in *F* for IBU.

## Discussion

In humans, IBU undergoes extensive phase I and phase II biotransformation to the main metabolites 2OH-IBU, COOH-IBU, and IBU-GlcA (Rainsford, 2009). The current study provides evidence that the same metabolites are formed in the conventional pig at different ages, suggesting the presence of similar biotransformation pathways. However, since 2OH-IBU, COOH-IBU, and IBU-GlcA were detected in pig plasma using a targeted quantitative method, it cannot be excluded that other IBU metabolites were formed as well. Untargeted analysis using UPLC-high resolution mass spectrometry (HR-MS) could reveal the identity of all IBU-derived metabolites, but only qualitative data would be generated as no analytical standards are commercially available besides the three metabolite standards used in the current study.

The plasma concentration of the three metabolites was always lower than the parent compound, IBU. IBU is known to be extensively metabolized in human as less than 1% of the IBU dose was recovered unchanged in 24-h urine of healthy adult volunteers (Tan et al., 2002). The low metabolite concentrations could possibly be due to the higher clearance of the metabolites compared to the clearance of IBU. Moreover, **Figure 2** shows that the elimination phase of the metabolites was parallel to the elimination of IBU. Consequently, the elimination of phase I and II metabolites of IBU is rate-limited by its formation.

No significant differences could be found in AUC of IBU after IV or PO administration. In combination with a high *F*, this might indicate limited to no first-pass biotransformation. Nevertheless, regarding the metabolites, the 4-week-old pigs showed a significantly lower AUC and *C*_max_ of 2OH-IBU after PO administration compared to IV. This might indicate that this age category could have a higher glucuronidation rate, or is subject to more pre-systemic conversion compared to the other age groups.

Interestingly, the metabolites AUC ratio for 2OH-IBU and COOH-IBU increased with age during the first 4 weeks of life, both after IV and PO administration. In human, these metabolites are formed by CYP2C8, CYP2C9, and CYP2C19. Due to the high homology (61.6–80.6%) between human and pig enzymes, the CYP2C subfamily might be involved in the biotransformation of IBU in pigs (Puccinelli et al., 2011; Millecam et al., 2018). The increase in AUC ratio for 2OH-IBU and COOH-IBU might be attributed to the increase of CYP2C enzyme abundancy with age. Indeed, Millecam et al. (2018) observed an increase in hepatic microsomal CYP2C protein abundancy from 3.84% at birth to 12.23% at 4 weeks of age, followed by a gradual increase to 20.62% at 6–7 months of age, using the same breed of conventional pigs as the present study. In human, both *in vitro* and *in vivo* data suggest an increase in CYP2C enzymes leading to an increased clearance of drugs metabolized by CYP2C (Treluyer et al., 1997; Koukouritaki et al., 2004). Moreover, Koukouritaki et al. (2004) found the *in vitro* enzyme activity of CYP2C9 to be greater in neonates from birth till 5 months of age compared to older children using diclofenac as substrate. The mephenytoin 4-hydroxylase activity of CYP2C19 in children aged 5 months till 10 years of age was also greater compared to younger and older children (Koukouritaki et al., 2004). In pigs, the *in vitro* tolbutamide 4-hydroxylase activity increased with age, but no decrease was observed (Millecam et al., 2018). In the current study, the 6- to 7-month-old pigs were characterized by a significant lower AUC ratio (IV) for 2OH-IBU and COOH-IBU compared to the 4-week-old pigs. This could be due to less formation of 2OH-IBU and COOH-IBU, a shift towards more and faster glucuronidation, formation of other still unknown metabolites, or other parameters such as volume of distribution (*V*_d_) and plasma protein binding. It should be noted, however, that the lower AUC ratio for 2OH-IBU and COOH-IBU in the 6- to 7-month-old pigs could also be attributed to the much higher AUC of IBU. This higher AUC might be a consequence of the lower *V*_d_ in the 6- to 7-month-old pigs compared to younger pigs, as has been demonstrated by Millecam et al., (2019). As can be seen from **Table 1**, the *C*_0_ of IBU after IV administration was indeed significantly higher compared to the other age categories, pointing towards a lower *V*_d_. This lower *V*_d_ could be due to an altered body composition of the 6- to 7-month-old pigs, having more than 20% body fat, or to a different plasma protein binding of IBU at this age (Warnants et al., 2006).

In contrast to phase I biotransformation, the glucuronidation capacity of IBU in pigs did not change with age as there were no observed age differences in AUC or AUC ratio after IV administration. However, the AUC ratio in the 8-week-old pigs after PO administration was significantly higher compared to IV. Moreover, the AUC ratio in the 8-week-old pigs was the highest compared to the other age groups, which might be due to a higher glucuronidation capacity or pre-systemic conversion. Nonetheless, the present study demonstrated that the UGT enzymes responsible for glucuronidation were already present in neonatal pigs. The maturation of pig UGT enzymes and generally all other pig phase II enzymes is still largely unknown. Van Peer et al. (2017) described an increasing UGT activity with age *in vitro* for Göttingen minipigs, using the UGT-Glo™ assay, which is consistent with several human studies, suggesting that glucuronidation capacity is more limited in neonates, infants, and children compared to adults (Krekels et al., 2012; Van Peer et al., 2017). Similar results were obtained after trimethoprim administration in pigs, where an increase in metabolic capacity for both phase I and II enzymes was observed (Gyrd-Hansen et al., 1984). This is in contrast with the results obtained in the current study indicating that maybe several UGT iso-enzymes are involved in the glucuronidation. Moreover, it could be possible that the involvement of the different UGT iso-enzymes in the glucuronidation of IBU changes during development in order to maintain a constant rate of glucuronidation. Nevertheless, it should be noted that only IBU-GlcA was determined in the current study and no glucuronidated phase I metabolites. Whether there is an increase in glucuronidated phase I metabolites with age should be further investigated. Reaching consensus about the enzyme ontogeny is difficult, since *in vitro* studies can focus on different levels, such as messenger ribonucleic acid (mRNA), enzymes, or proteins, in different test systems, as, for example, microsomes or hepatocytes. Results obtained after *in vivo* studies should be the most representative to unravel the ontogeny, as a lot of factors can influence the biotransformation of a drug (Krekels et al., 2012). Since the glucuronidation ratio of IBU did not change much with age in the current study, it is hypothesized that the phase II enzymes involved are already present in neonatal pigs and do not change with age, or there is maturation of phase II enzymes, but other enzymes can take over the metabolic capacity, so the net result stays the same.

The in vivo metabolism of IBU in growing conventional pigs as determined in the present study could only be discussed descriptively, since the exact fraction of the IBU dose biotransformed into the different metabolites is currently unknown. More extensive research should make it possible to build a compartmental PK model taking age and maturation into account to fully describe the PK of IBU and its metabolites.

In conclusion, IBU was shown to undergo phase I and phase II biotransformation in conventional pigs to the same metabolites as observed in human. Age did have a significant effect on the oxidative metabolism of IBU. However, age did not seem to affect the glucuronidation capacity of IBU in pigs. These results might suggest the potential use of the growing conventional pig as a preclinical animal model to evaluate the biotransformation of drugs, but should be further validated using other substrate drugs. In particular, regarding phase II glucuronidation, more research is required, in both humans and pigs.

## Ethics Statement

Care and use of the animals were in full compliance with the national and European legislation on animal welfare and ethics, and the study was approved by the ethical committee of the Faculties of Veterinary Medicine and Bioscience Engineering of Ghent University (EC2016/105) (Anonymous, 2010; Flemish Government, 2017).

## Author Contributions

JM, MD, and SC contributed to the conception and design of the study. JM performed the animal trials, as well as bioanalytical, pharmacokinetic, and statistical analysis. SB aided in the bioanalytical method development and validation. JM wrote the first draft of the manuscript. MD contributed in the PK analysis. All authors contributed to manuscript revision, and read and approved the submitted version.

## Funding

This study was funded by the Agency for Innovation by Science and Technology in Flanders and the Agency for Innovation and Entrepreneurship in Flanders (IWT, SB141427). Phoenix^®^ software was provided by Certara through their Centers of Excellence programme.

## Conflict of Interest Statement

The authors declare that the research was conducted in the absence of any commercial or financial relationships that could be construed as a potential conflict of interest.
