# Supplementary material for: In Vivo Metabolism of Ibuprofen in Growing Conventional Pigs: A Pharmacokinetic Approach
Source: Front Pharmacol. 2019 Jun 27;10:712. doi: 10.3389/fphar.2019.00712 (PMC6610244; doi:10.3389/fphar.2019.00712)
Supplement: Supplementary file 1 [file Table_1.docx]

**Supplemental Table S1**: Results of the evaluation of linearity (goodness-of-fit (gof), correlation coefficient (r)), limit of quantification (LOQ) and limit of detection (LOD) for 2-hydroxyibuprofen (2OH-IBU), carboxyibuprofen (COOH-IBU) and ibuprofen acyl-β-D-glucuronide (IBU-GlcA) in pig plasma.

| Analyte | calibration range (ng/mL) | gof (%) | r | LOQ (ng/mL) | LOD (ng/mL) |
| --- | --- | --- | --- | --- | --- |
| 2OH-IBU | 20-3000 | 4.49 | 0.999 | 20 | 0.59 |
| COOH-IBU | 20-3000 | 3.15 | 0.999 | 20 | 0.59 |
| IBU-GlcA | 10-600 | 4.85 | 0.998 | 10 | 0.27 |
| Acceptance criteria for linearity: gof < 10% and r > 0.99 | | | | | |

**Supplemental Table S2**: Results of the within- and between-run precision and accuracy evaluation for the analysis of 2-hydroxyibuprofen (2OH-IBU), carboxyibuprofen (COOH-IBU) and ibuprofen acyl-β-D-glucuronide (IBU-GlcA) in pig plasma.

|  |  | **Within-day (n = 6)** | | **Between-day (n = 3x3)** | |
| --- | --- | --- | --- | --- | --- |
|  | theoretical concentration (ng/mL) | accuracy (%) | precision (RSD, %) | accuracy (%) | precision (RSD, %) |
| 2OH-IBU | 200 | 7.0 | 5.2 | -5.5 | 4.9 |
|  | 750 | 1.6 | 5.1 | 1.6 | 6.0 |
|  | 2000 | -15.8 | 3.1 | -6.6 | 8.6 |
| COOH-IBU | 200 | 3.2 | 6.4 | -7.6 | 2.2 |
|  | 750 | -1.2 | 6.2 | -0.1 | 5.8 |
|  | 2000 | -10.3 | 3.5 | -3.5 | 3.0 |
| IBU-GlcA | 40 | -8.7 | 13.5 | -3.6 | 6.3 |
|  | 150 | -3.9 | 9.5 | 4.4 | 13.8 |
|  | 400 | -10.2 | 7.6 | -2.6 | 5.6 |
| RSD: relative standard deviation.  Acceptance criteria for within- and between-day accuracy for all three metabolites: -20% to +10%  Acceptance criteria for within-day precision (RSD_max_) for all three metabolites: 10%, except for IBU-GlcA at the level of 40 ng/mL RSD_max_ is 15%  Acceptance criteria for between-day precision (RSD_max_) for 2OH-IBU and COOH-IBU: 200 ng/mL: 20.4%; 750 ng/mL: 16.7% and 2000 ng/mL: 14.4%  Acceptance criteria for between-day precision (RSD_max_) for IBU-GlcA: 40 ng/mL: 26.0%; 150 ng/mL: 21.3% and 400 ng/mL: 18.4% | | | | | |

carboxyibuprofen

carboxyibuprofen

hydroxyibuprofen-d6

2-hydroxyibuprofen

ibuprofen acyl-β-D-glucuronide

ibuprofen acyl-β-D-glucuronide

Time

**Supplemental Figure S1**: Chromatograms of a plasma sample collected at 45 min after intravenous administration of 5 mg/kg BW ibuprofen to a 8-week-old pig. If present, both the identification and quantification trace are demonstrated. Following precursor ion → product ion transitions are demonstrated from top to bottom: carboxyibuprofen: mass-to-charge ratio (*m/z*) 235.1 → 191.2 (collision energy (CE) 10 eV) for identification and *m/z* 235.1 → 72.9 (CE 15 eV) for quantification; hydroxyibuprofen-d6: *m/z* 227.1 → 183.3 (CE 9 eV); 2-hydroxyibuprofen: *m/z* 221.4 → 177.2 (CE 8 eV); ibuprofen acyl-β-D-glucuronide: *m/z* 381.2 → 193.0 (CE 10 eV) for identification and *m/z* 381.2 → 112.9 (CE 15 eV) for quantification.
